# An Exploration of Heart Failure Risk in Breast Cancer Patients Receiving Anthracyclines with or without Trastuzumab in Thailand: A Retrospective Study

**DOI:** 10.3390/clinpract11030064

**Published:** 2021-08-02

**Authors:** Jukapun Yoodee, Aumkhae Sookprasert, Phitjira Sanguanboonyaphong, Suthan Chanthawong, Manit Seateaw, Suphat Subongkot

**Affiliations:** 1 Department of Pharmaceutical Care, Faculty of Pharmacy, Chiang Mai University, Chiang Mai 50200, Thailand; jukapun.y@cmu.ac.th; 2 The College of Pharmacotherapy of Thailand, Nonthaburi 11000, Thailand; phitjira.s@ubu.ac.th; 3 Medical Oncology Unit, Department of Medicine, Faculty of Medicine, Khon Kaen University, Khon Kaen 40002, Thailand; aumkhae@yahoo.com; 4 Division of Pharmacy Practice, Faculty of Pharmaceutical Sciences, Ubon Ratchathani University, Ubon Ratchathani 34190, Thailand; manit.s@ubu.ac.th; 5 Division of Clinical Pharmacy, Faculty of Pharmaceutical Sciences, Khon Kaen University, Khon Kaen 40002, Thailand; suthch@kku.ac.th

**Keywords:** anthracyclines, breast cancer, cardiotoxicity

## Abstract

Anthracycline-based regimens with or without anti-human epidermal growth factor receptor (HER) 2 agents such as trastuzumab are effective in breast cancer treatment. Nevertheless, heart failure (HF) has become a significant side effect of these regimens. This study aimed to investigate the incidence and factors associated with HF in breast cancer patients treated with anthracyclines with or without trastuzumab. A retrospective cohort study was performed in patients with breast cancer who were treated with anthracyclines with or without trastuzumab between 1 January 2014 and 31 December 2018. The primary outcome was the incidence of HF. The secondary outcome was the risk factors associated with HF by using the univariable and multivariable cox-proportional hazard model. A total of 475 breast cancer patients were enrolled with a median follow-up time of 2.88 years (interquartile range (IQR), 1.59–3.93). The incidence of HF was 3.2%, corresponding to an incidence rate of 11.1 per 1000 person-years. The increased risk of HF was seen in patients receiving a combination of anthracycline and trastuzumab therapy, patients treated with radiotherapy or palliative-intent chemotherapy, and baseline left ventricular ejection fraction <65%, respectively. There were no statistically significant differences in other risk factors for HF, such as age, cardiovascular comorbidities, and cumulative doxorubicin dose. In conclusion, the incidence of HF was consistently high in patients receiving combination anthracyclines trastuzumab regimens. A reduced baseline left ventricular ejection fraction, radiotherapy, and palliative-intent chemotherapy were associated with an increased risk of HF. Intensive cardiac monitoring in breast cancer patients with an increased risk of HF should be advised to prevent undesired cardiac outcomes.

## 1. Introduction

Breast cancer is considered one of the most significant health problems among women worldwide [1,2]. Anthracycline-based chemotherapy regimens have been effectively shown to improve disease-free survival (DFS) and overall survival (OS) in patients with early and locally advanced breast cancer [3,4,5]. These regimens have also improved progression-free survival (PFS) in patients with metastatic disease [4,6]. In breast cancer patients with human epidermal growth factor receptor 2 (HER2) status positive, supplementing trastuzumab subsequently after systemic chemotherapy significantly improves DFS, OS, and PFS in HER2 positive breast cancer both in adjuvant and in metastatic settings [7,8,9,10].

Although the efficacy of breast cancer treatment is improved when anthracyclines are combined with anti-HER2 agents, cardiac dysfunction, especially heart failure (HF), remains the most prominent adverse event of these therapies [10]. In general, anthracyclines potentiate cardiac myocyte death by inducing double-strand DNA breaks and increasing radical oxygen species, leading to increased oxidative stress and subsequent irreversibly damaged cardiac myocytes. Cardiac dysfunction owing to anthracyclines is observed after administering the first dose of chemotherapy, and it is related to a cumulative dose effect [11]. In addition, trastuzumab inhibits HER2 signaling pathway in cardiac myocytes leading to loss of contractility, and this effect is reversible upon discontinuing treatment [12]. Recent clinical practice guidelines, including the American Society of Clinical Oncology (ASCO) and the European Society for Medical Oncology (ESMO), specified that patients treated with high-dose anthracyclines (e.g., doxorubicin ≥ 250 mg/m^2^) or lower-dose anthracyclines (e.g., doxorubicin < 250 mg/m^2^) in combination with anti-HER2 therapy (e.g., trastuzumab) are at increased risk of HF [13,14].

A previous report has revealed that the incidence of HF after breast cancer treatment was increased in patients receiving anthracycline-based regimens (1.1–1.4 fold) compared with those receiving non-anthracycline-based regimens [15]. In addition, the incidence of HF was augmented (2.0–7.0 fold) when concomitantly administering anthracycline-containing regimen with trastuzumab [7,16]. Conversely, the incidence of HF was lower when trastuzumab was given as a sequential therapy [17]. Recent large population-based cohort studies in Denmark [18] and Taiwan [19] have found a lower incidence of HF following breast cancer treatment in Asian populations than European populations.

In a recent study, several specific risk factors related HF in breast cancer patients treated with anthracyclines with or without trastuzumab have been reported [13]. Advanced age, postmenopausal status, mediastinal radiotherapy, high body mass index (BMI), borderline low left ventricular ejection fraction (LVEF), and cardiovascular comorbidities (e.g., hypertension and diabetes) have increased the risk of cardiac dysfunction mediated by anthracyclines with or without trastuzumab [13,18].

Nevertheless, systematically scientific evidence of cardiac dysfunction due to anthracycline-based regimens in breast cancer is not well investigated in Thailand. Some previously reported risk factors of HF in the literature remain controversial and await to be confirmed. Therefore, this study was aimed to investigate the incidence and factors associated with HF in breast cancer patients treated with anthracyclines with or without trastuzumab.

## 2. Materials and Methods

### 2.1. Study Design and Data Collection

This single-center retrospective cohort study was conducted at Srinagarind Medical Center, Faculty of Medicine, Khon Kaen University, Khon Kaen, Thailand, the largest university-affiliated cancer center in the north-eastern region of Thailand. The electronic medical records (EMRs) of all breast cancer patients treated with anthracyclines with or without trastuzumab between 1 January 2014 and 31 December 2018 were reviewed. Data from the EMRs were retrieved and screened for patient characteristics, treatment interventions (e.g., breast cancer treatment protocols), underlying disease, and adverse effects management, with an emphasis on HF management. Anthracycline doses were converted to doxorubicin-equivalent doses to compare the effects on cardiac dysfunction. The study design was approved by the Center for Ethics in Human Research, Khon Kaen University, Khon Kaen, Thailand (approval number: HE621307).

### 2.2. Patients and Treatments

The inclusion criteria were as follows: (1) age ≥ 18 years, (2) diagnosis of breast cancer (any stage), (3) history of at least one cycle of any anthracycline-based regimen with or without trastuzumab, (4) documentation of baseline LVEF assessment using echocardiography before the starting of drug therapy, and (5) baseline LVEF ≥ 50%. The exclusion criteria were as follows: (1) patients with a confirmed history of HF before breast cancer diagnosis, (2) uncontrolled hypertension defined as systolic blood pressure  ≥140 mmHg and/or diastolic blood pressure  ≥90 mmHg in patients receiving antihypertensive treatment, and (3) breast cancer treatment during pregnancy. All patients used anthracycline-based regimens for breast cancer therapy. If patients were confirmed as HER2-positive, trastuzumab was administered as a subsequent therapy after completing the chemotherapy regimen for 1 year [3,4].

### 2.3. Outcomes

The primary outcome was the incidence of HF after initiating chemotherapy. All patients were followed from the starting date of chemotherapy to the first onset of HF. The time to the event (HF) was the interval between the date-of chemotherapy started to the date of the first HF event occurred. Observations were censored if the patients were lost to follow-up or had not yet experienced HF by the study end date (time to last follow-up or 31 September 2019), whichever came first. The HF diagnosis was confirmed individually by a cardiologist and oncologist based on LVEF and clinical HF symptoms recorded in the EMR. The defined criteria for HF were a decrease in LVEF >10% from baseline to <50%; for patients treated with trastuzumab, the additional criteria included a decrease in LVEF >16% along with HF symptoms [13,16]. Echocardiography was performed in all patients before administering the anthracycline-based regimens. In patients treated with trastuzumab, echocardiography was performed every 3 months after initiating trastuzumab through the completion of the 1-year treatment course. Symptomatic HF was assessed according to the New York Heart Association (NYHA) classification of class III or higher and subsequently confirmed using the Common Terminology Criteria for Adverse Events (CTCAE) version 5.0 for HF grade III or decreased LVEF grade III or higher [14,20,21]. The secondary outcomes were clinical characteristics of HF, time to develop HF after treatment initiation, and factors associated with HF in breast cancer patients treated with anthracyclines with or without trastuzumab. The early onset HF was defined as the development of HF during 12 months after the completion of chemotherapy, and the late-onset HF was defined as the development of HF later than 12 months after chemotherapy has been completed.

### 2.4. Statistical Analyses

Descriptive statistics were used to analyze baseline demographics. Two groups of continuous variables were compared using ‘Student’s *t*-tests or Mann–Whitney U-tests, as appropriate. Fisher’s exact test was used to compare categorical variables between groups and an independent *t*-test or one-way ANOVA for continuous variables. The incidence of HF was reported as a percentage and calculated per 1000 person-years. Univariable and multivariable Cox proportional hazards models were used to assess the risk factors associated with HF. The potential risk factors including age, baseline LVEF, cardiovascular-related comorbidities, radiotherapy, palliative-intent therapy, trastuzumab, and cumulative anthracycline dose were adjusted in the multivariable analysis. Schoenfeld’s global test method was performed test proportional hazards assumption [22]. Statistical significance was determined using a two-sided test with a *p*-value of <0.05. STATA, version 15 (StataCorp LP, College Station, TX, USA), was used for statistical analysis and data management. 

## 3. Results

### 3.1. Baseline Characteristics

A total of 475 patients were included in this study. The median time to follow-up was 2.88 (interquartile range [IQR], 1.59–3.93) years. Most patients were female (99.6%).

The mean age at breast cancer diagnosis was 52.4 ± 10.7 years and not statistically significantly different between the HF and non-HF groups (56.7 ± 11.2 vs. 53.2 ± 10.8, *p* = 0.115). The mean baseline LVEF was 68.6 ± 6.5%. The mean cumulative anthracycline dose was 247.5 ± 39.7 mg/m^2^. The proportion of radiation therapy was 54.7% and slightly higher in HF group but not statistically significantly different from non-HF groups (80% vs. 53.9%, *p* = 0.063). Cardiac function (e.g., left ventricular end-diastolic diameter (LVEDD) and valvular heart function) before chemotherapy treatment were normal in all patients. The baseline characteristics of patients with HF and non-HF events were similar, except for the proportion of HER2-positive patients (73.3% vs. 39.1%, *p* = 0.005), LVEF before chemotherapy (65.3 ± 6.1% vs. 68.7 ± 6.5%, *p* = 0.046), LVEDD before chemotherapy (48.7 ± 4.9 mm vs. 43.8 ± 4.6 mm, *p* = 0.003), and trastuzumab use (60.0% vs. 17.6%, *p* < 0.001) (Table 1).

### 3.2. Incidence of HF after Breast Cancer Treatments

**Figure 1:**
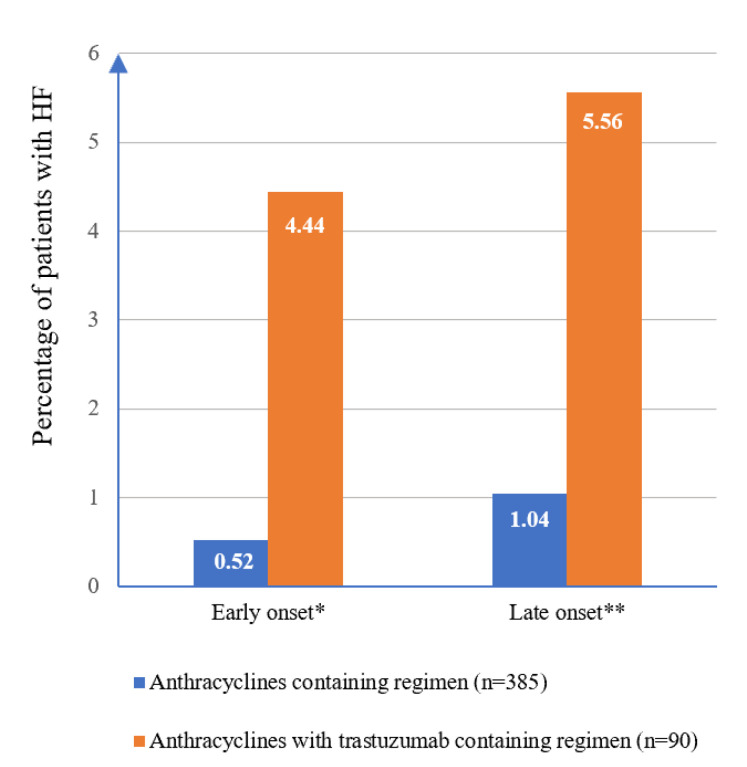
Incidence of HF according to onset (*n* = 475). * Early onset refers to HF occurs during the year after the completion of chemotherapy. ** Late onset refers to HF occurs later than a year after chemotherapy has been completed.

The overall incidence of HF among breast cancer patients treated with anthracyclines with or without trastuzumab was 3.2%, with an incidence rate of 11.1 per 1000 person-years. Patients treated with anthracycline-based regimens and trastuzumab therapy had a higher incidence and incidence rate of HF than those treated with anthracyclines alone (10.0% vs. 1.6% and 39.7 vs. 5.3 per 1000 person-years, respectively; both *p* < 0.001). The incidence of early and late-onset HF in patients who received anthracycline-based regimens and trastuzumab therapy was higher than anthracycline alone (Figure 1).

There were no reports of HF in patients with metastatic disease treated with anthracycline-based regimens with trastuzumab therapy or trastuzumab alone. The clinical characteristics of HF included symptomatic HF only in seven patients (1.5%) and symptomatic HF with a significant decrease in LVEF in eight patients (1.7%). The incidence and incidence rate of HF are shown in Table 2.

### 3.3. Factor Associated with HF on Univariable and Multivariable Analysis

Univariable analysis indicated that the factors significantly increased the risk of HF were trastuzumab use (HR, 7.36; 95% CI: 2.62–20.69; *p* < 0.001), and baseline LVEF of <65% (HR: 3.48; 95% CI: 1.26–9.59; *p* = 0.016). In the multivariate analysis, treatment in palliative-setting (HR, 7.06; 95% CI: 1.53–32.23; *p* = 0.012), trastuzumab use (HR, 5.46; 95% CI: 1.67–17.83; *p* = 0.005), radiotherapy (HR, 5.03; 95% CI: 1.26–22.46; *p* = 0.034), and baseline LVEF < 65% (HR, 3.89; 95% CI: 1.36–11.10; *p* = 0.011) increased the risk of HF. There were no statistically significant differences with respect to age, cardiovascular-related comorbidities, and cumulative dose of doxorubicin, as shown in Table 3. The proportional hazard assumption test showed no evidence of violation for all the tested models (*p* = 0.154).

## 4. Discussion

HF is the most common cardiac toxicity related to anthracycline-based chemotherapy with or without trastuzumab. To the best of our knowledge, this is the largest study to report the incidence and factors associated with HF after breast cancer treatment in Thailand.

### 4.1. Incidence of HF after Initiating Chemotherapy

In this study, the overall incidence of HF was higher than that of the previous large observational studies from Denmark [18] and Taiwan [19] (3.2% vs. 1.12–1.15%) but consistent with a previous incidence report from Thailand of 5.6% [23]. The greater incidence in our study might have been due to different diagnostic criteria for HF, cardiovascular comorbidities, and advanced-stage breast cancer.

As the current study used a more strictly diagnostic criteria for HF, including a decrease of >10% in LVEF from baseline to <50% in patients treated with anthracyclines alone [14,18,19,23]. The additional diagnostic criteria, including decreased LVEF >16% with HF symptoms, were also applied with subsequent trastuzumab treatment [14]. These strictly diagnostic criteria might have influenced the clinician to be more proactively involved in HF symptom identification and monitoring. In contrast to the recent studies from both Denmark [18] and Taiwan [19] whereby the diagnostic criteria of HF symptoms appeared to be less rigid. In addition, cardiovascular comorbidities and advanced-stage disease at baseline might have affected the HF incidence outcome report following breast cancer therapy in this current study [8,18,19]. Evidently, the result from a large randomized controlled trial (NCCTG-N9831 and NSABP-B31 study) revealed that patients with low cardiovascular comorbidities and early-stage breast cancer demonstrated a lower incidence of HF [8,24].

Pharmacodynamically, synergistic effect of anthracyclines and anti-HER2 increases HF risk as shown in both early and late onset of cardiac toxicity [18]. The incidence of HF among patients receiving anthracycline-based regimens and trastuzumab therapy in this currently were also higher in both early and late-onset, which was consistent with the result from the previous observational study [18].

### 4.2. Factors Associated with HF in Breast Cancer Patients

Our study explored the potential risk factors associated with HF and factors resulting in a significantly increased risk of HF included radiotherapy, trastuzumab use, palliative-intent treatment, and patients with baseline LVEF of <65%. This finding was similar to those of previous studies [8,25].

The effect of radiation therapy increased the relative risk of cardiac events, and this risk was higher among patients with pre-existing heart disease [26,27]. In patients treated with anthracyclines and anti-HER2 combination with radiotherapy, the risk of HF appeared to be more intensified [28]. Unsurprisingly, trastuzumab use in our study was a strong risk factor universally known as anti-HER2 induced cardiac dysfunction. It negatively affects cardiac myocytes by disrupting the HER2 signaling pathway leading to dysregulation of toxic substances and loss of contractility of cardiac myocytes [12,13]. Many studies also indicated that the risk of HF was augmented when combining trastuzumab with anthracyclines [7,8,9]. Our study also found that patients with advanced-stage breast cancer receiving palliative-intent treatment had an increased risk of HF. This association could have been related to compromised performance status, multiple disease-related complications, and the inability to tolerate aggressive treatments [13,14]. Previous randomized controlled trials uncovered those patients with palliative-intent treatment were at increased risk of HF [10]. Our study observed similar results to those of the NSABP B31 study, whereby the effects of high-risk cardiovascular disease increased the risk of HF [25]. In addition, the baseline LVEF of less than 65% could have increased the risk of HF (HR:3.89; 95%CI:1.36–11.10; *p* = 0.016), which was consistent with the result of the large randomized controlled trial NSABP B31 study whereby patients with LVEF <65% had been associated with an increased risk of HF (HR:6.72; 95%CI: 2.67–16.92; *p* < 0.001) [25].

Theoretically, the HF risk from anthracycline-based regimens with or without trastuzumab appears to relate to the lifetime cumulative doxorubicin dose (specifically, a total dose >450 mg/m^2^) [13]. In this study, the mean cumulative anthracycline dose was 247.5 mg/m^2^, lower than the reported threshold in the literature, particularly in patients receiving trastuzumab [13,24]. Therefore, the association between cumulative doxorubicin dose and HF could not be demonstrated. According to the ASCO and ESMO clinical practice guidelines, the risk of HF increased with high-dose anthracyclines (doxorubicin ≥250 mg/m^2^) or lower-dose anthracyclines (doxorubicin <250 mg/m^2^) combined with anti-HER2 therapy (trastuzumab) [13,14]. To detect anthracycline-related cardiac dysfunction at a lower cumulative dose of doxorubicin (100–200 mg/m^2^), echocardiographic global longitudinal strain (GLS) has become an innovative clinical standard with high sensitivity and specificity to help identify the incidence and risk factors associated HF [29]. In the previous observational study, the increase of age was not associated with the risk of HF [18,19]. Our study observes similar results to both studies. Nonetheless, this would require a larger sample size with multivariable-adjusted analysis for an effect to be statistically significant and meaningful.

### 4.3. Strengths and Limitations

This study highlighted the clinical aspect where specific patient characteristics and risk factors could have affected the development of HF in breast cancer patients undergoing treatment with anthracycline-based regimens with or without trastuzumab. Although this was a single-center retrospective study, it was the largest cohort study of breast cancer patients receiving anthracyclines in Thailand [30]. In addition, the time to follow-up in this study was 2.88 years, which was longer than the duration seen in a previous study (3.5 months) [30]. A longer follow-up period would be required to identify sub-chronic cardiac dysfunction owing to anthracyclines, which generally occur 2–10 years after treatment initiation [14,31]. The results from this study may have emphasized the importance of active screening and closed monitoring in patients with a high risk of HF (a low LVEF and trastuzumab use) who are undergoing anthracycline-based therapy to prevent undesirable cardiac outcomes [14].

This study had some limitations. First, in the studied practice settings, echocardiography is mandated for all patients prior to anthracycline-based therapy, but it is optional for subsequent trastuzumab therapy due to resource constraints. Instead, a clinical evaluation of HF signs and symptoms is performed in all patients. This could have led to an underestimation of the true incidence of HF. Second, several confounders may have affected the outcome evaluation of this retrospective study; some residual risk factors could remain in this study, e.g., dietary and lifestyle factors. Although multivariable analyses were used to adjust for potential risk factors, we could not adjust for dietary and lifestyle factors because these records were not available in the EMR. Finally, the sample size in this study might not have been enough power to evaluate the effects of treatment-related modifiers (e.g., trastuzumab use, radiotherapy, cumulative anthracycline dose, and palliative-intent treatment) and non-treatment-related modifiers (e.g., age- and cardiovascular-related comorbidities) on HF risk according to the ASCO/ESMO guidelines [13,14]. A larger sample size will be required to perform subgroup analysis.

### 4.4. Study Implications and Future Research

Our findings suggest that cardiac monitoring in breast cancer patients treated with anthracyclines with or without anti-HER2 therapy is recommended to prevent HF. Further studies are needed to evaluate the relationship between genetic predisposition and cardiac toxicity from anthracyclines with or without anti-HER2 agents in Thai and Asian populations.

## 5. Conclusions

In conclusion, the incidence of HF in breast cancer patients treated with anthracyclines with or without trastuzumab was 3.2%. Patients with a baseline LVEF <65%, received radiotherapy, palliative-intent treatment, and treatment with anthracyclines and trastuzumab as sequential therapy appeared to have an increased risk of HF. Intensive Cardiac function monitoring should be performed early in high-risk patients to prevent HF.

## Figures and Tables

**Table 1:** Baseline and treatment characteristics (*n* = 475).

Characteristic	Total (*n* = 475)	HF Event (*n* = 15)	Non-HF Event (*n* = 460)	*p*-Value
Gender (female), *n* (%)	473 (99.6)	15 (100.0)	458 (99.6)	1.000
Age at diagnosis (years), mean ± SD (95% CI)	52.4 ± 10.7 (51.4–53.3)	56.7 ± 11.2 (50.5–62.8)	52.3 ± 10.8 (51.3–52.3)	0.115
Age (years), *n* (%)				
<65	411 (86.5)	11 (73.3)	400 (87.0)	0.130
≥65	64 (13.5)	4 (26.7)	60 (13.0)	
BMI (kg/m^2^), mean ± SD (95% CI)	24.5 ± 4.1 (24.1–24.8)	25.8 ± 3.8 (23.7–27.9)	24.4 ± 4.1 (24.1–24.8)	0.190
Disease stage, *n* (%)				
I	43 (9.0)	2 (13.3)	41 (8.9)	0.379
II	225 (47.4)	6 (40.0)	219 (47.6)	
III	160 (33.7)	4 (26.7)	156 (33.9)	
IV	47 (9.9)	3 (20.0)	44 (9.6)	
Postmenopausal status, *n* (%)	305 (64.2)	10 (66.7)	295 (64.1)	1.000
Hormone receptor status, *n* (%)				
ER-positive	301 (63.4)	8 (55.3)	293 (63.7)	0.425
PR-positive	231 (48.6)	6 (40.0)	225 (48.9)	0.603
HER2-positive, *n* (%)	177 (37.3)	11 (73.3)	166 (39.1)	0.005
LVEF before chemotherapy (%), mean ± SD (95% CI)	68.6 ± 6.5 (68.0–69.1)	65.3 ± 6.1 (61.8–68.7)	68.7 ± 6.5 (68.1–69.3)	0.046
LVEDD before chemotherapy (mm), mean ± SD (95% CI)	44.0 ± 4.7 (43.5–44.4)	48.7 ± 4.9 (45.5–51.8)	43.8 ± 4.6 (43.3–44.3)	0.003
LA size before chemotherapy (mm), mean ± SD (95% CI)	31.1 ± 5.3 (30.1–31.7)	36.8 ± 4.6 (33.8–39.9)	30.9 ± 5.2 (30.4–31.5)	0.002
Cardiovascular-related comorbidities, *n* (%) ^a^	107 (22.5)	5 (33.3)	102 (22.2)	0.345
Medication for pre-existing cardiovascular comorbidities, *n* (%) ^b^	89 (18.7)	4 (26.7)	85 (18.5)	0.497
Radiation therapy, *n* (%)	260 (54.7)	12 (80.0)	248 (53.9)	0.063
Chemotherapy, *n* (%)				0.177
Curative-intent ^c^	428 (90.1)	12 (80.0)	416 (90.4)	
Palliative-intent	47 (9.9)	3 (20.0)	44 (9.6)	
Hormone therapy use, *n* (%) ^d^	288 (60.6)	7 (46.7)	281 (61.1)	0.290
Cumulative anthracycline dose (mg/m^2^), mean ± SD (95% CI)	247.5 ± 39.7 (243.8–251.1)	240.2 ± 37.8 (219.1–261.0)	247.7 ± 39.8 (244.0–251.4)	0.466
Trastuzumab use, *n* (%)	90 (19.0)	9 (60)	81 (17.6)	<0.001

^a^ Cardiovascular comorbidities refer to at least one of the following comorbidities: hypertension, type 2 diabetes mellitus, dyslipidaemia, atrial fibrillation, ischemic heart disease, and valve disease. ^b^ Medication for pre-existing cardiovascular comorbidities refers to at least one of the following medications: CCBs, statins, ACEIs/ARBs, ASA, and beta-blockers. ^c^ Curative-intent treatment: neoadjuvant and adjuvant therapy. ^d^ Hormone therapy includes tamoxifen, letrozole, and anastrozole. Abbreviations: ACEI, angiotensin-converting enzyme inhibitor; ARB, angiotensin II receptor blocker; ASA, acetylsalicylic acid; BMI, body mass index; CCB, calcium-channel blocker; CI, confidence interval; ER, estrogen receptor; HER2, human epidermal growth factor receptor 2; HF, heart failure; LA, left ventricular; LVEF, left ventricular ejection fraction; LVEDD, left ventricular end-diastolic diameter; PR, progesterone receptor; SD, standard deviation.

**Table 2:** Incidence of heart failure (*n* = 475).

Incidence of Heart Failure	Overall (*n* = 475)	Anthracycline Alone (*n* = 385)	With Trastuzumab (*n* = 90)	*p*-Value
Clinical HF ^a^	15 (3.2)	6 (1.6)	9 (10.0)	<0.001
Curative-intent treatment ^b^	12	3	9	
Palliative-intent treatment	3	3	0	
Incidence rate (per 1000 person-years)	11.1	5.3	39.7	<0.001
Median (IQR) time between treatment initiation and HF (days)	392 (238–681)	613 (140–1478)	380 (273–437)	0.020
Clinical characteristics of HF				
Significant decrease in LVEF ^c^ and symptomatic HF ^d^	8 (1.7)	3 (0.8)	5 (5.6)	0.622
Symptomatic HF only ^d^	7 (1.5)	3 (0.8)	4 (4.4)	

^a^ Clinical HF: CTCAE version 5.0 in HF grade III or decreased LVEF grade III or higher [20]. ^b^ Curative-intent treatment: neoadjuvant and adjuvant therapy. ^c^ Significant decrease in LVEF: absolute decrease in LVEF >10% from baseline to <50% in patients treated with anthracyclines alone; when treated with anti-HER2 agents, absolute decrease in LVEF >16% [3]. ^d^ Symptomatic HF: New York Heart Association classification of class III or higher and confirmed using the CTCAE version 5.0 for HF grade III or higher [3,20]. Abbreviations: CTCAE, Common Terminology Criteria for Adverse Events; HER2, human epidermal growth factor receptor 2; HF, heart failure; IQR, interquartile range; LVEF, left ventricular ejection fraction.

**Table 3:** Univariable and Multivariable analysis of risk factors associate with HF (*n* = 475).

Risk Factor	Univariable Analysis			Multivariable Analysis *		
	HR	95%CI	*p*-Value	HR	95%CI	*p*-Value
Age						
<65 years	1.00			1.00		
≥65 years	2.68	0.85–8.43	0.093	3.55	0.95–13.21	0.059
Baseline LVEF						
≥65%	1.00			1.00		
<65%	3.48	1.26–9.59	0.016	3.89	1.36–11.10	0.011
Cardiovascular comorbidities ^a^						
No	1.00			1.00		
Yes	2.03	0.69–5.97	0.198	1.33	0.39–4.58	0.65
Radiotherapy						
No	1.00			1.00		
Yes	3.11	0.88–11.03	0.079	5.03	1.26–22.46	0.034
Treatment in palliative setting						
No	1.00			1.00		
Yes	3.62	0.99–13.18	0.051	7.06	1.53–32.23	0.012
Cumulative doxorubicin						
<250 mg/m^2^	1.00			1.00		
≥250 mg/m^2^	0.61	0.19–1.93	0.401	1.21	0.33–4.50	0.772
Trastuzumab use						
No	1.00			1.00		
Yes	7.36	2.62–20.69	<0.001	5.46	1.67–17.83	0.005

^a^ Cardiovascular comorbidities refer to at least one of the following comorbidities: hypertension, type 2 diabetes mellitus, dyslipidemia, atrial fibrillation, ischemic heart disease, and valve disease. * Multivariable analysis by Cox proportional hazard model adjusted for age, baseline LVEF, cardiovascular-related comorbidities, radiotherapy, palliative-intent therapy, trastuzumab, and cumulative anthracycline dose. Abbreviations: CI, confidence interval; HR, hazard ratio; LVEF, left ventricular ejection fraction.

## Data Availability

No additional data were generated in the study.
